# Construction and application of computerized risk assessment model for supply chain finance under technology empowerment

**DOI:** 10.1371/journal.pone.0285244

**Published:** 2023-05-04

**Authors:** Bo Huang, Wei Gan

**Affiliations:** 1 School of Business, Guangdong Polytechnic of Science and Techenology, Zhuhai, China; 2 School of Business, Macau University of Science and Technology, Taipa, Macau, China; Jazan University Faculty of Computer Science, SAUDI ARABIA

## Abstract

This study seeks to assist small and medium enterprises break free of the constraints of the conventional financing model and lessen the supply chain finance risks they face. First, the supply chain financial business model and credit risk are analyzed, followed by a discussion of the application principle of blockchain in the control of supply chain financial credit risk. The next topic up for discussion is the emancipation of individuals and the application of financial technology toward the management of financial risk in supply chains. In the final stage of the development of the computerized risk assessment model, the Fuzzy Support Vector Machine (FSVM) is optimized, and the effectiveness and efficiency of risk classification are enhanced by introducing a variable penalty factor C. To test the efficacy of the C-FSVM risk assessment model, the Chinese auto sector is used as the study’s object. According to the results of the study, the C-FSVM model has a classification accuracy of 96.35% for the entire sample, 96.45% for credible firms, and 95.34% for default enterprises. The training time of the C-FSVM model is 473.9s, which is far lower than the SVM and FSVM models’ training times of 1631.6s and 1870.2s. In summary, the C-FSVM supply chain financial risk assessment model is effective and has great application value in banking practice.

## Introduction

There is a network chain structure made up of SMEs that collaborate upstream and downstream, linked by the risk platform of supply chain finance and the cooperation platform of the industrial supply chain. Supply chain finance in this instance evaluates the upstream and downstream of the supply chain based on the structure of supply chain transactions and the comprehension of operational risks, with the aid of the credits of core firms or the self-compensation of online merchants. True to its name, risk management is fundamental to supply chain finance [1–3]. Its business strategy relies heavily on the supply chain’s central companies, which have both high qualifications and strong credit, driving upstream and downstream businesses with comparatively low credit qualifications to undertake financing using the credit of the central enterprises. Simply put, a substantial volume of transaction data provided by suppliers and core firms must be processed through risk control before credit is extended.

The overall financial infrastructure for the dynamic expansion of the technology-enabled supply chain must seek to enter industries based on strategic needs. It is not viable to construct a large, all-encompassing, cross-functional platform in the business-to-business sector. It is possible to fine-tune and efficiently manage the supply chain structure by breaking it down into smaller parts based on the high-level design [4,5]. Supply chain finance has the potential to play a unique role in bolstering the growth of small and micro companies by providing a suite of complete financial services, including financing, settlement, and cash management, to SMEs both upstream and downstream in the supply chain. Improved quality and efficiency of supply chain financial services (SCFSs) for SMEs can be anticipated as a result of the rapid evolution of a number of emerging technologies, including big data, artificial intelligence, blockchain, and fifth-generation communication technology [6–8]. The qualities of blockchain technology are a perfect fit for the monetary operations of supply chains. Accounts receivable can be certified on the chain when they are issued by the central company, transmitted among suppliers at all levels, and subsequently financed by financial institutions due to the immutable and highly reliable nature of the data stored in blockchains [9]. Accounts receivable can be tracked back to their original source, guaranteeing that all contracts arising from such transactions are genuine.

A growing number of fintech firms are specializing in the supply chain industry, and they’re doing so by offering businesses digital SCFSs and technological solutions based on blockchain [10–12]. Companies in the scientific and technical sectors employ "blockchain + electronic certificate" technology to streamline their supply chains, reduce overhead, and maximize return on investment. Blockchain’s permitted encryption technology is also being used by some businesses to bring previously unverifiable volumes of offline transactions online while still maintaining consumer confidentiality. In light of the aforementioned literature, this study examines the potential of blockchain technology for controlling financial credit risks in supply chains from a blockchain viewpoint. First, the blockchain-based supply chain financial risk (SCFR) management principle is clarified. Second, the empowerment and application of financial technologies in SCFR management are analyzed. In the final stage of the development of a computerized risk assessment model, the Fuzzy Support Vector Machine (FSVM) is enhanced, and the effectiveness and efficiency of risk categorization are enhanced by incorporating a variable penalty factor C.

## Materials and methods

### Supply chain finance business model and credit risk

Large and medium-sized businesses are frequently well-established, financially stable, and operate on a large scale. When it comes to funding, commercial banks and other financial institutions typically favor large and medium-sized businesses, ignoring the requirements of SMEs. By using the credit of core firms to reactivate corporate inventories and accounts receivable, the SCFR management model helps SMEs resolve their financing problems, lowers the cost of financing for corporations, and improves the operating efficiency of funds [13,14].

**Fig 1:**
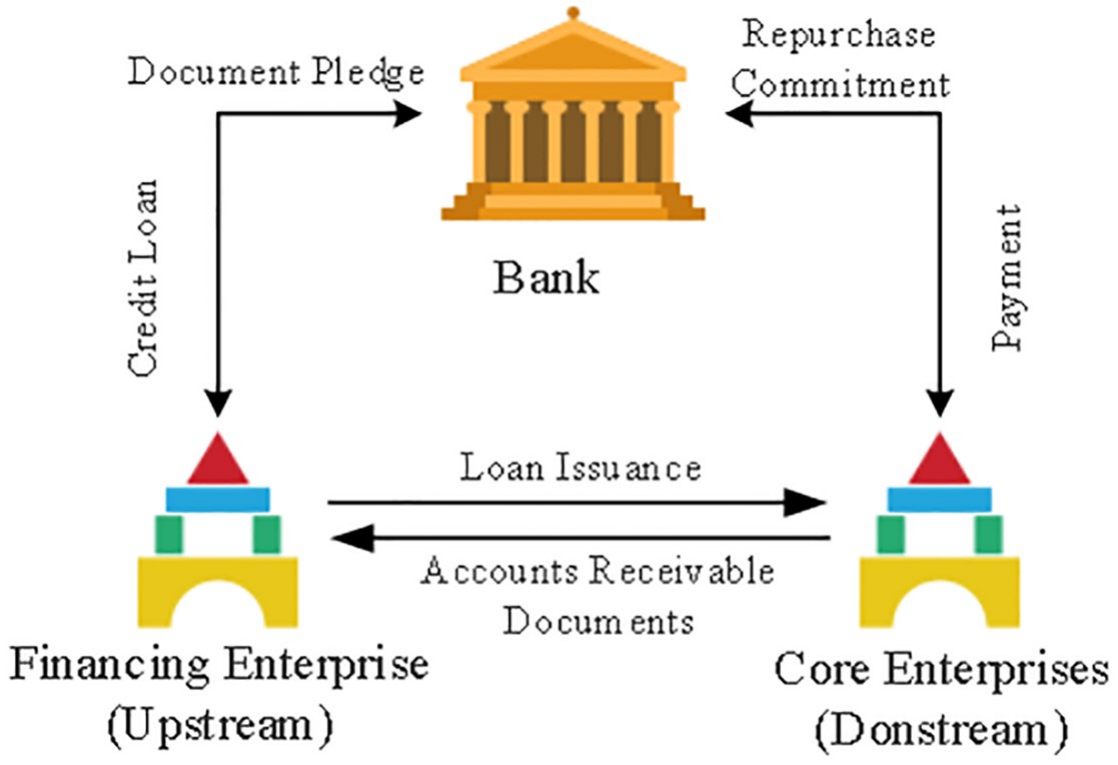
Accounts receivable financing model.

Accounting receivables are pledged as collateral for business loans so that companies can fund their operations. Fig 1 depicts the financial model. It is common practice for the two businesses to agree on the settlement method of goods first and payment later following the signing of a contract, as there is a particular cycle for the core business’s sales payment. That’s why businesses that need a steady flow of cash to keep investing in production and operations keep accounts receivable on their books. A business in need of financing may be able to secure a loan by pledging its accounts receivable as collateral. For the capital-hungry business to be able to repay the loan, the parent company issues directives and/or guarantees and agrees to transfer the proceeds of any sales to the lender. After considering the application, the agency makes a decision about whether or not to approve the loan.

**Fig 2:**
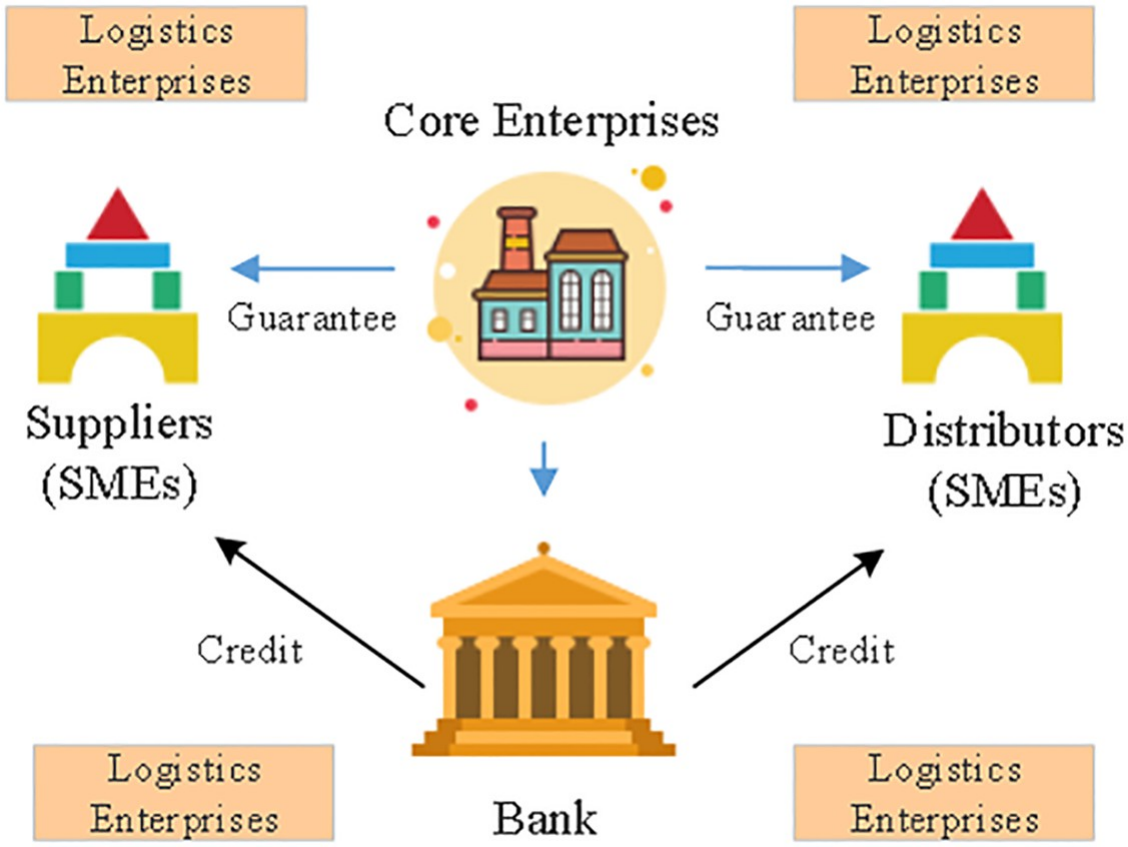
Connection between the four subfields of supply chain finance.

Buyers farther down the supply chain can use confirmed warehouse finance to apply for loans on the platform to pay upstream core suppliers in advance of future products deliveries. Concurrently, the supplier agrees to repurchase any items that aren’t picked up, and will transfer the right to do so to a finance model managed by banks. Financing warehouses are a type of inventory financing in which a company uses its stock as collateral in a loan arrangement with a bank. Therefore, the finance warehouse service may provide high-level logistics services for companies, resolve financing issues for SMEs, close the cash flow gap in business operations, and boost supply chain efficiency [15–17]. The monetary links between the various actors in the supply chain are depicted in Fig 2. Risk management concerns are just one example of the difficulties that have arisen during the evolution of supply chain financing. The current state of supply chain finance development is imperfect, making it difficult to manage both external risks like macroeconomic cycles and the financial environment and internal risks like business operating risks and financial hazards.

The current paradigm for managing the financial risks inherent in supply chains is predicated mostly on the management and monitoring of key firms. Unfortunately, there are two major flaws with this model. On the one hand, the lack of thorough pre-loan investigation, loan supervision, and post-loan management might lead central enterprises to blindly give credit guarantees for upstream and downstream enterprises based on various social networks. On the other hand, fraud involves a conspiracy between businesses to fake supply contracts. There is a significant increase in supply chain risk because of the existing absence of appropriate tools to deal with information asymmetry among chain companies and collusion fraud among chain businesses [18]. It is also evident that the governance and risk management procedures pertaining to supply chain finance might stand to be enhanced. Disputes about who owns certain items have become commonplace due to the high volume of goods moving through the supply chain. Moreover, the supply chain is crucial to the functioning of supply chain finance. The vast majority of companies throughout the supply chain are SMEs, as opposed to core businesses. There are issues with their corporate governance process, technical force, and personnel turnover. Organizational risk is increased by massive and erratic business operations, and financial statements are notoriously unreliable when it comes to business processing.

### Principle of the blockchain for financial risk control of supply chain

The data in a blockchain cannot be altered and verification is straightforward. The information from one data block is used to construct the next block. Checking the final block’s function value is sufficient for confirming all accounting. Under the current technical framework, blockchain may be broken down into three distinct types: public chain, alliance chain, and private chain. Table 1 displays the specific comparison. SMEs’ loan repayment behavior can be broken down into two categories: compliance and non-compliance. However, the factors that lead to non-compliance are not well understood. Even though a non-recourse account receivable financing model exists, most financial institutions would choose a recourse model instead due to the greater security it provides. Blockchain technology can perform transaction endorsement and guarantee verification beyond the third party and transfer the credit of core firms layer by layer, allowing SMEs to raise financing and increasing the income of the entire supply chain [19,20]. Thanks to the blockchain, all supply chain actors may easily view the same data without incurring any additional costs as a result.

**Table 1:** Comparison of three types of blockchain.

	Public chain	Alliance chain	Private chain
Participant	Free entry for anyone	Specific groups of people (with agreement)	individual
Trust mechanism	Proof of work	Collective endorsement	Self-endorsed
Degree of centralization	Decentralized	Weak centralization	Strong centralization
Are the incentives essential?	Need	Optional	Unnecessary
Advantage	Credit self-establishment	Efficiency improvement	Traceable and transparent
Application scenario	Bitcoin	Liquidation	Audit

**Fig 3:**
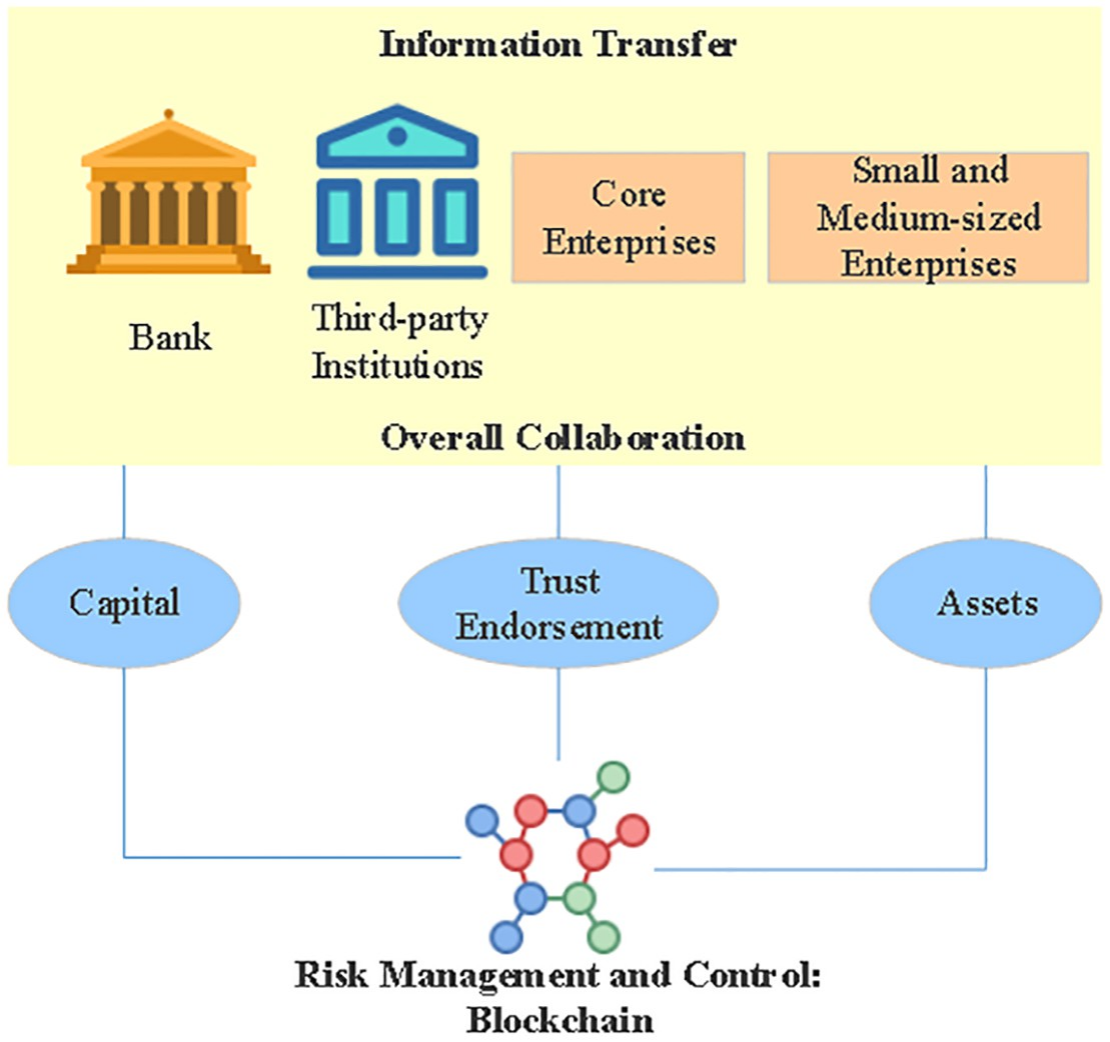
Principles of SCFR control based on blockchain technology.

The consortium technique is used by the blockchain-based supply chain financial system in all related situations. Users include financial institutions like financing banks, core merchants, and third-party logistics providers, and supply chain members including upstream and downstream shops acting as nodes in the network. Safe, efficient, and consistent with standard business practice are only some of the features of the six-tiered blockchain technology that characterizes the alliance link between the public and private chains. Alliance chains in supply chain finance use contracts and other techniques to create a blockchain that facilitates trust and consensus amongst participating enterprises and institutions. Fig 3 reveals the principle of SCFR control based on blockchain technology.

Credit penetration of the supply chain financial system is made possible by blockchain technology, which also alleviates the burden of high financing costs for intermediaries. Two distinct functions are fulfilled by the blockchain. In order to resolve the credit financing issues faced by secondary suppliers, it is necessary to first check the rights of the core enterprise, which includes verifying and certifying the authenticity and validity of the complete bill.

To effectively transmit credit along the supply chain, reduce cooperation costs, and increase performance efficiency, this ecology of trust allows the credit of core enterprises (bills, credit lines, or confirmation of payables) to be converted into digital warrants, while smart contracts are used to prevent performance risks [21]. When digital warrants are anchored on the blockchain, funds may be split and circulated between upstream and downstream firms via smart contracts, dramatically increasing the speed of funds and resolving the financing challenges and expensive financing of SMEs.

Commercial banks get access to the supply chain in terms of financial financing thanks to the trust endorsement connection between the upstream core firms and the downstream SMEs, using the accounts receivable financing model as an example. Insight into how a large company C earns the trust of a small company S can be gained by applying evolutionary game theory to this process. If the core enterprise agrees to guarantee the financing of SMEs, its expected income can be written as Eq (1).


$$U_{c1}=y\left(S_{c}-C_{1}\right)+\left(1-y\right)\left(-C_{1}-C_{c}\right)$$ (1)

A company’s predicted profit is given by Eq (2) if the main business does not agree to guarantee the funding of SMEs.


$$U_{c2}=0$$ (2)

Then, Eq (3) presents the core business’ projected profit under the combined guarantee and non-guarantee strategy.


$$U_{c}=xU_{c1}+\left(1-x\right)U_{c2}=xU_{c1}$$ (3)

Eq (4) expresses the replication dynamic equation of the core enterprise’s choice of financing.


$$F\left(x\right)=\frac{dx}{dt}=x\left(U_{c1}-U_{c}\right)=x\left(1-x\right)\left(yS_{c}-yC_{c}-C_{1}-C_{c}\right)$$ (4)

Functions representing the income expectations of small and medium-sized enterprises (SMEs) considering financing, non-financing, and mixed strategies are as follows.


$$U_{s1}=x\left(S_{c}-C_{2}\right)\left(1-x\right)\left(-C_{2}-C_{s}\right)$$ (5)

$$U_{s2}=0$$ (6)

$$U_{s}=yU_{d1}+\left(1-y\right)U_{s2}=yU_{s1}$$ (7)

A corresponding replication dynamic equation for SMEs to select financing is given by Eq (8).


$$F\left(y\right)=\frac{dy}{dt}=y\left(U_{s1}-U_{s}\right)=y\left(1-y\right)\left(xS_{s}-yC_{s}-C_{s}-C_{s}\right)$$ (8)

Combine Eqs (4) and (8) to form a system of equations:

$$\left\{\begin{array}{l} F\left(x\right)=\frac{dx}{dt}=0\\ F\left(y\right)=\frac{dy}{dt}=0 \end{array}\right.$$ (9)

Further solution can be obtained according to Eqs (10) and (11).


$$x_{1}=0,x_{2}=0,x^{*}=\frac{C_{2}+C_{s}}{S_{s}+C_{c}}$$ (10)

$$y_{1}=0,y_{2}=0,y^{*}=\frac{C_{1}+C_{c}}{S_{c}+C_{c}}$$ (11)

Ultimately, five game behaviors can be obtained, namely 0(0, 0), A(0, 1), B(1, 0), C(1, 1), and D($\frac{C_{2}+C_{s}}{S_{c}+C_{c}},\frac{C_{1}+C_{c}}{S_{c}+C_{c}}$).

**Fig 4:**
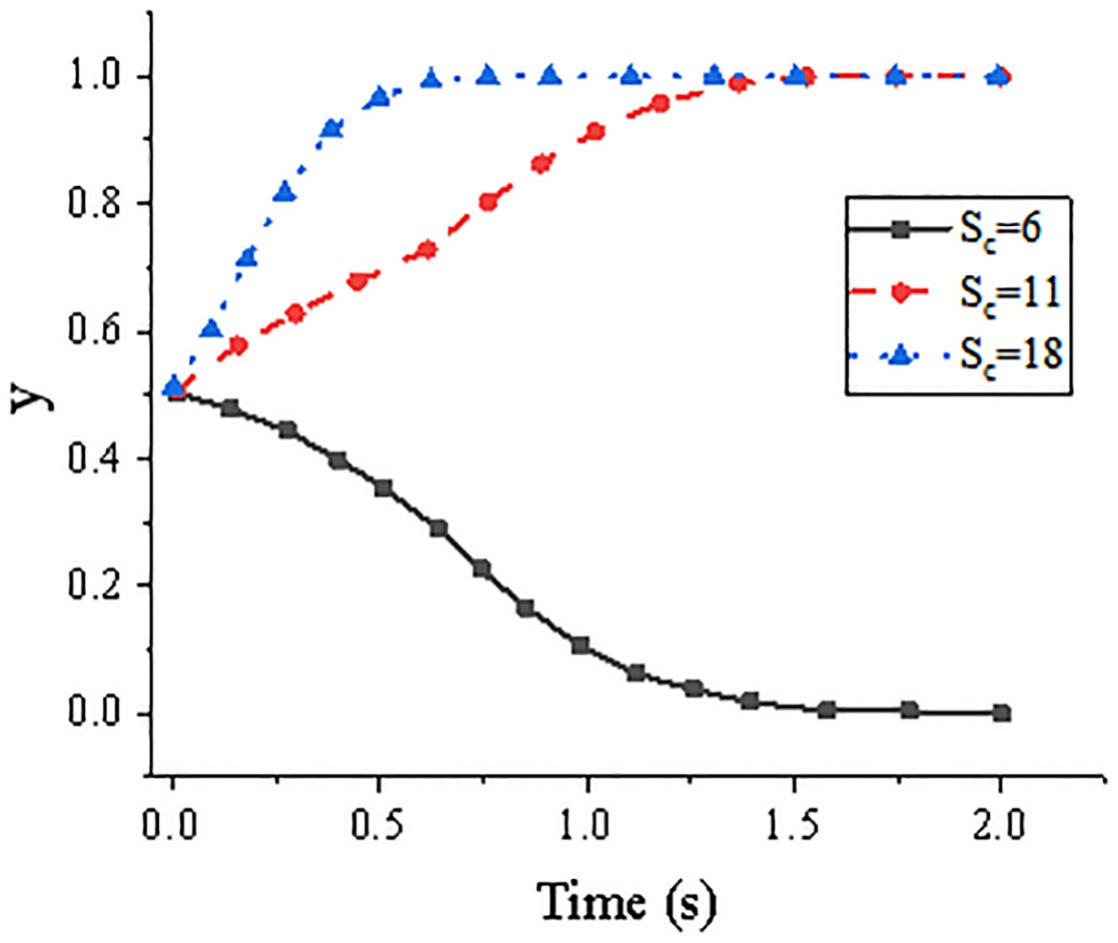
Evolution trend of the system over time under the changing value of *S*_*c*_.

**Fig 5:**
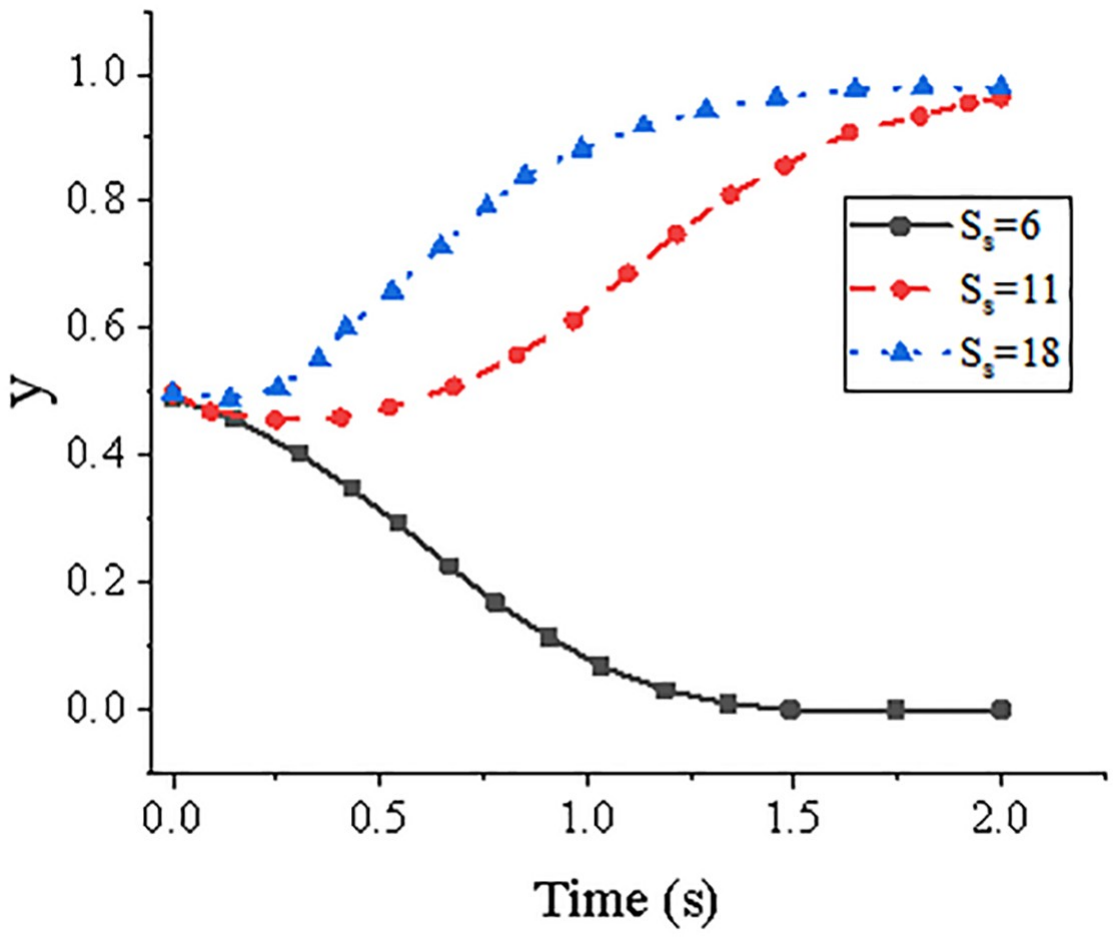
Evolution trend of the system over time under the changing value of *S*_*s*_.

Given the initial value of the system (0.5, 0.5). Fig 4 illustrates the system’s evolution trend over time for the years 6, 11, and 18. Fig 5 depicts the system’s evolution over time at ages 6, 11, and 18, respectively. It has been observed that a more positive attitude towards guaranteeing on the part of core firms can help advance the growth of all enterprises across the whole supply chain, creating a win-win situation. It is easy for there to be a lack of desire on both sides to seek guarantee and financing collaboration if the core firms in the supply chain give guarantees for SMEs, especially if the cost of the guarantee process increases as more procedures are engaged.

### Empowerment and application of the SCFR control technology

Supply chain finance works best in sectors with wide market spaces, dispersed upstream and downstream markets, standardized production goods, constant demand, and modest price changes. As a result, commercial banks place a premium on sectors with established markets and high levels of maturity, such as the petrochemical, coal, electricity, and non-ferrous metals sectors, because of the resource features of these sectors. Supply chain financing has expanded into new areas, propelled by the proliferation of Internet-enabled devices and services, such as in the computer communications, medical, agricultural, light industrial, retail, and other sectors.

The growth of supply chain financing generally and the promotion of digitization and capitalization through increased transparency and efficiency are all factors that contribute to the emergence of digital supply chain finance [22,23]. Connecting the Internet with the physical manufacturing supply chain and the financial system is essential to financing the digital supply chain. It completely permeates the industrial chain by utilizing the fundamental data and application scenarios to achieve universal connectivity and swift monetary flow. There is a high barrier to entry when it comes to securing funding for SMEs because of the challenges associated with data collection and data quality verification. A sophisticated financial risk control system is constructed via the Internet of Things (IoT) and artificial intelligence technologies. A modular zoom camera that can be swapped out is part of the hardware design for the IoT sensors that are deployed everywhere; this improves the efficiency with which data is collected and processed across a wide variety of industrial settings. This allows for data processing at the edge to be more efficient and less resource-intensive thanks to the enhanced software and hardware system architecture.

By establishing an accurate risk control and early warning system, businesses can fortify client institutions to supply financial institutions with more authentic and dependable data during the pre-loan approval process and guarantee the approval effect. The team will execute sensor fusion processing on the aforementioned multi-channel data and link enterprise data with generic risk control indicators using foundational technologies like smart IoT and edge computing to boost the indicators’ credibility, reach, and responsiveness. The direct coupling of systems like enterprise resource planning and software-as-a-service, as well as the availability of data interfaces, ensures the data’s veracity against the backdrop of monetary technology’s emancipatory potential; real-time monitoring and timely warning are conducted based on the comprehensive credit granting model for transaction data and external data; the IoT enables the linking of digital information with physical objects, hence lowering the price tag associated with product monitoring [24].

### Construction of the SCFR assessment model based on FSVM

In supply chain finance, the credit risk of SMEs comes from both objective breach of contract and moral hazard and the objective breach of contract and moral hazard of their counterparties (leading enterprises in the supply chain). On the one hand, debt repayment capacity is influenced by the financial health and management of SMEs and their counterparties; on the other hand, the supply chain’s future prospects and competitive climate. Stability in the cooperative relationship between SMEs and leading firms and the credit status (including financial performance and credit level) of both parties should have an impact on the moral hazard of both. As a result, the financial position and credit level of supply chain counterparties, the number of supply chain partnerships, and the level of supply chain development are all factors that influence SMEs’ credit risk. In addition, the emergence of third-party credit complicates the assessment of credit risk in supply chain finance. If commercial banks wish to evaluate the credit risk of certain SMBs, they must analyze the supply chain, which includes the SMBs and their partners [25].

**Fig 6:**
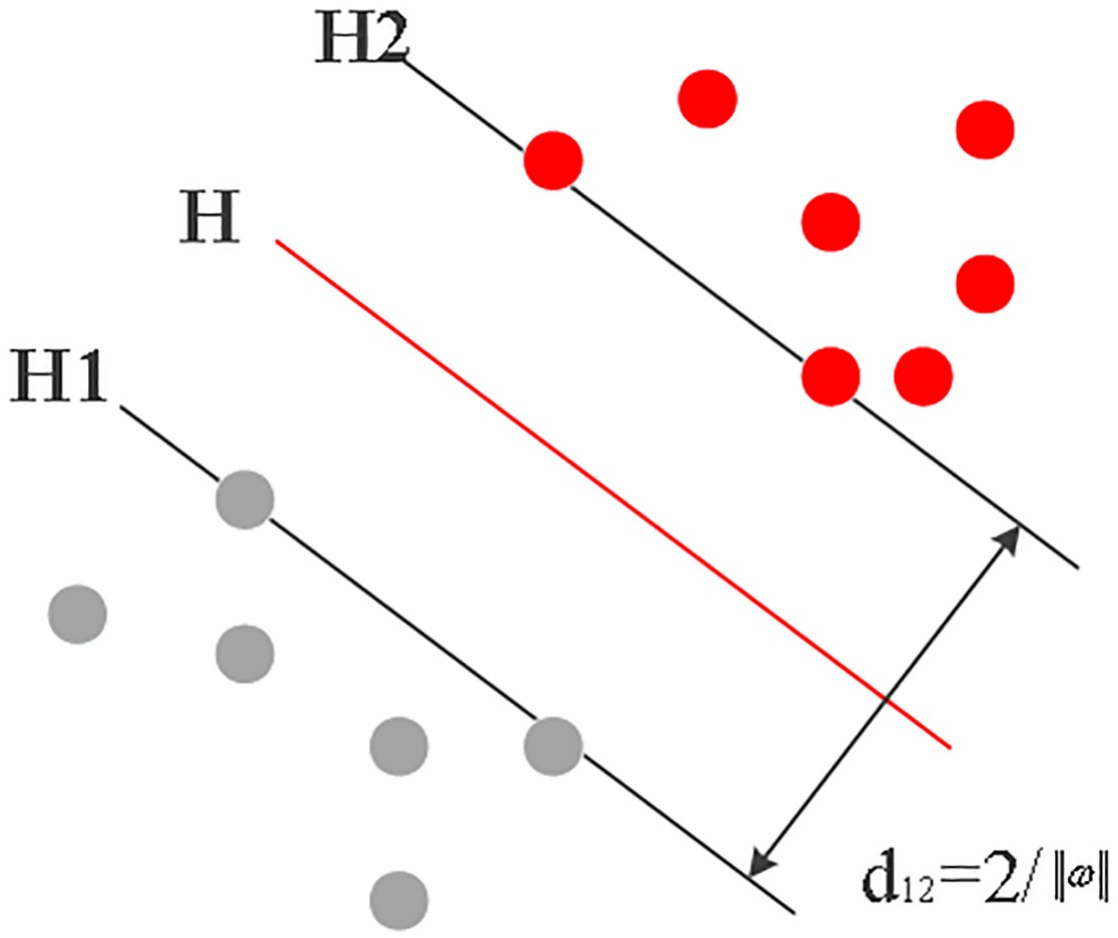
Optimal classification hyperplane.

The neural network and SVM-based classification model have a remarkable capacity for identifying credit risk. Consequently, this study introduces the support vector machine model for assessing the credit risk of SMBs in the supply chain. SVM, a machine learning algorithm based on kernels, excels at addressing nonlinear, separable classification problems. SVM’s learning technique, by constructing the multidimensional decision-making optimal classification hyperplane, may accomplish the ideal separation of two categories of data with reduced empirical risk (Fig 6). Hyperplanes H1 and H2 in Fig 6 are the ideal ones since they are perpendicular to the H surface and cut through the two categories of samples that are most in close proximity to the H surface. The distance *d*_12_ between them is the classification interval, which can be written as Eq (12).

$$d_{12}={\text{min}\;}_{\left\{x_{i}:y_{i}=1\right\}}\frac{\left|\omega \cdot x_{i}+b\right|}{\left\|\omega \right\|}+{\text{min}\;}_{\left\{x_{i}:y_{i}=-1\right\}}\frac{\left|\omega \cdot x_{j}+b\right|}{\left\|\omega \right\|}$$ (12)

In Eq (12), *ω* stands for the feature space dimension, and *b* refers to the undetermined scalar parameter.

When assigning credit to newly collected data, SVM often uses the most recently classified samples as the default, reliable ones. It is challenging to execute an exact credit risk evaluation via SVM in the supply chain financing mode because the target financing firm is not entirely credible or defaulting, and there are frequently various indications used for evaluation. The fuzzy link between credible samples and default samples is described by the membership degree determination function and the fuzzy relationship matrix in the FSVM theory, which are used in the FSVM state analysis method. This process continues until the membership degree matrix has been set. For fuzzy reasoning, a membership function is chosen. The search for the best solution to the objective function can be seen as a way to solve the optimal classification surface problem.

$$\left\{\begin{array}{l} \text{min}\;J\left(\omega \right)=\frac{1}{2}{\left\|\omega \right\|}^{2}+C\sum_{i=1}^{n}s_{i}\varepsilon_{i}\\ s.t.\;y_{i}\left(\omega \cdot x_{i}+b\right)+\varepsilon_{i}\geq 1 \end{array}\right.$$ (13)

In Eq (13), *C* signifies the penalty factor. The optimal discriminant function of FSVM can be written as Eq (14).

$$f\left(x\right)=\text{sgn}\;\left(\omega^{*}\cdot x+b^{*}\right)$$ (14)

wherein:

$$\omega^{*}=\sum_{i=1}^{n}\alpha_{i}^{*}y_{i}\;x_{i}$$ (15)

$$b^{*}=y_{i}-\sum_{i=1}^{n}y_{i}\alpha_{i}^{*}\left(x_{i}\cdot x_{j}\right)$$ (16)

In light of the fact that the bank should punish defaulting customers more severely, knowing which customers are likely to go into default is more crucial than knowing which customers are reliable. This study builds on the FSVM model by introducing a new variable penalty component for model optimization. Finally, the empirical risk and confidence interval can be balanced with the help of the non-negative penalty factor C, which allows SVM model classification to incur the least amount of structural risk possible. Therefore, the value of the penalty factor should satisfy Eq (17).

$$\left|f\left(x\right)\right|\leq \left|\sum_{i=1}^{n}a_{i}K\left(x,x\prime \right)\right|\leq \sum_{i=1}^{n}\left|a_{i}\right|\cdot K\left(x,x\prime \right)\leq \sum_{i=1}^{n}C\cdot K\left(x,x\prime \right)$$ (17)

After incorporating the penalty variable, the quadratic programming issue is stated as Eq (18).

$$\left\{\begin{array}{l} \text{min}\;J\left(\omega \right)=\frac{1}{2}{\left\|\omega \right\|}^{2}+\sum_{i=1}^{n}C_{i}s_{i}\varepsilon_{i}\\ s.t.\;y_{i}\left(\omega \cdot x_{i}+b\right)+\varepsilon_{i}\geq 1 \end{array}\right.$$ (18)

The ideal classification function can be derived by picking the proper kernel function and variable penalty factor *C*_1_, as shown in Eq (19).

$$f\left(x\right)=\text{sgn}\;\left(\omega^{*}\cdot x+b^{*}\right)$$ (19)

### Application analysis of SCFR assessment

This study chooses China’s automobile manufacturing industry as its research object in order to precisely analyze the C-FSVM risk assessment model developed here. There are 82 different businesses involved in the auto industry; ten are core firms that produce cars in the center of the supply chain, and the remaining 78 are SMEs that focus on sales and provide things like engines and software for cars. Shenzhen Stock Exchange provides annual reports and information disclosure data for each firm, while the "China Enterprise Financial Information Analysis Database" supplies the quantitative data for the sample.

Principal component analysis on the SCFR assessment indices is performed using the Factor procedure in SPSS software. Parameters are optimized using a 5-fold cross-check procedure, which yielded C1 = 20 and C2 = 750 for the penalty factors of credible and default samples, respectively. Classification outcomes are recorded and compared with preexisting classification data, and both the training and test samples are randomly picked for testing a total of five times.

## Result and discussion

### Chain financial risk assessment based on the C-FSVM model

**Fig 7:**
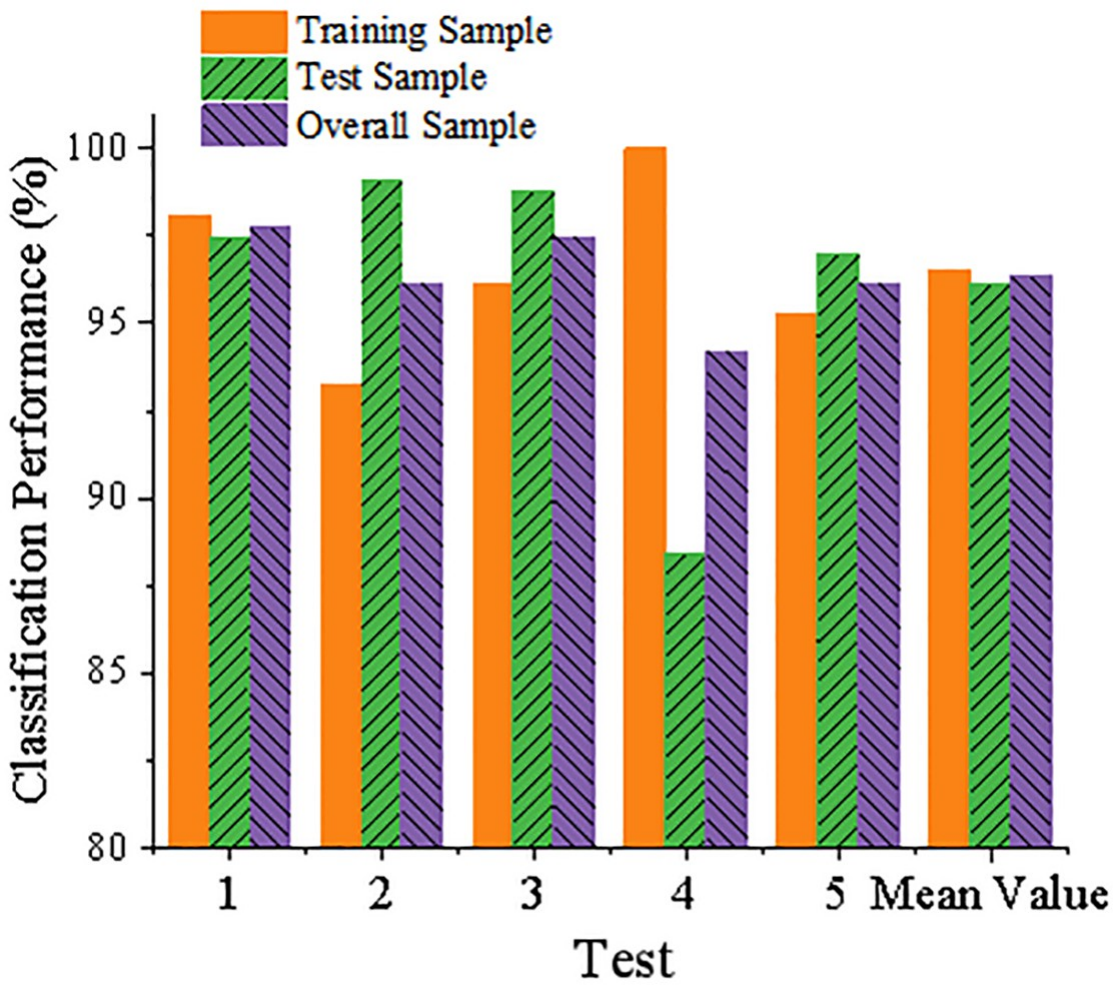
Statistical results of sample classification accuracy.

The main component f is input for model training in Matlab, and the classification results for the training samples are acquired. Next, the trained model is put to the test by inserting the test samples into it. Rates of type I error (for credible users) and type II error (for default customers) are displayed in Table 2 for the test sample, training sample, and total sample, respectively. Fig 7 shows the classification accuracy of the sample. The total sample classification accuracy of the C-FSVM model is found to be 96.35%, with a type I error rate of 3.55% and a type II error rate of 4.66%. This translates to a recognition accuracy rate of 96.45% for the trustworthy enterprise and a rate of 95.34% for the default firm. This empirical result shows that the performance of the C-FSVM SCFR assessment model is better, and the type II error rate that will cause greater losses is less than 5%, which meets the requirements of banks.

**Table 2:** Error rate statistics of C-FSVM model.

Test number	Test sample		Training samples		Total sample	
	Type I error rate	Type II error rate	Type I error rate	Type II error rate	Type I error rate	Type II error rate
1	6.67%	8.33%	3.33%	4.55%	4.44%	5.88%
2	0.00%	8.33%	0.00%	9.09%	0.00%	8.71%
3	6.67%	8.33%	3.33%	0.00%	5.00%	4.16%
4	6.67%	0.00%	6.67%	4.55%	6.67%	2.27%
5	0.00%	0.00%	3.33%	4.55%	1.66%	2.27%
Average	4.00%	5.00%	3.33%	4.55%	3.55%	4.66%

### Contrastive analysis of SCFR assessment models

**Fig 8:**
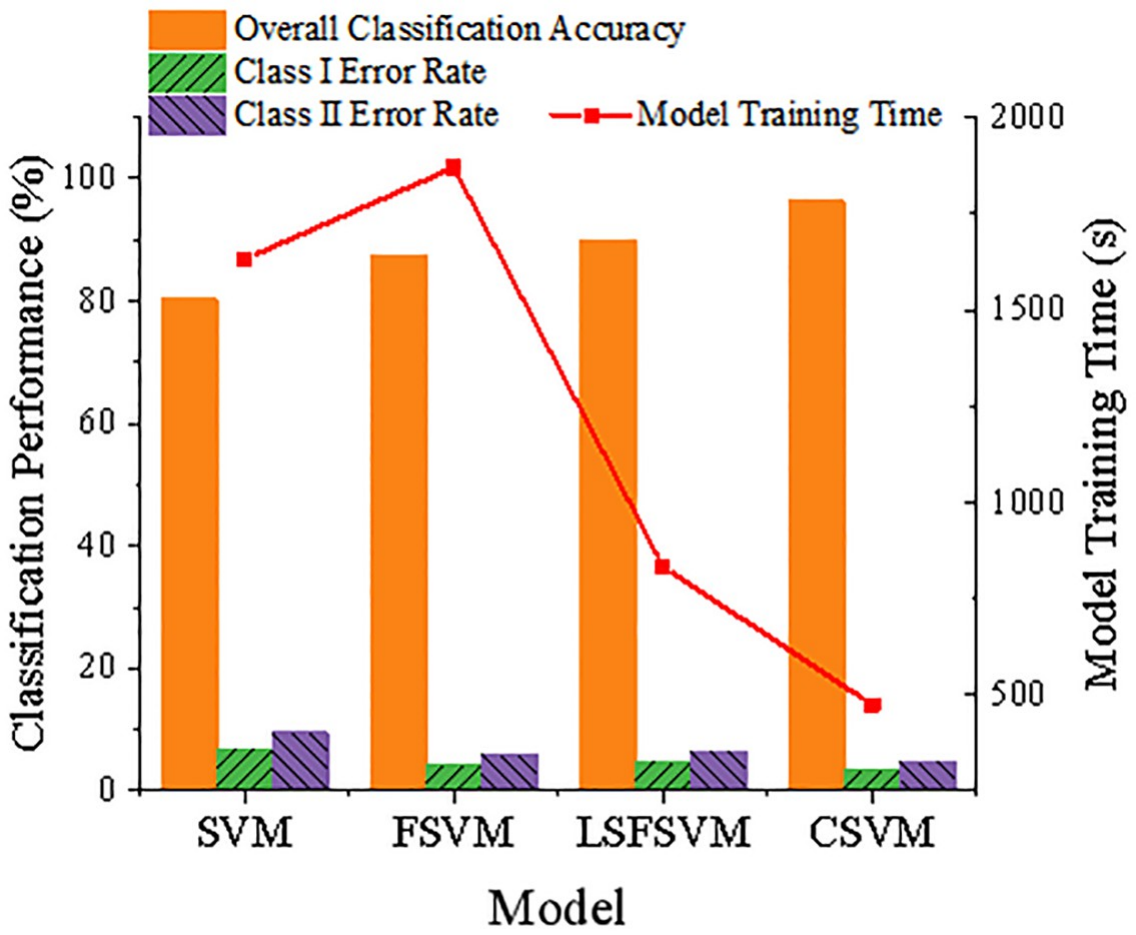
Comparative analysis results of risk assessment models.

Classification accuracy and model training time are two areas where the C-FSVM model stands out from its competitors, the SVM, the FSVM, and the LSFSVM. Fig 8 provides the specific comparison results. It can be seen that the training time of the C-FSVM model is 473.9s, which is far lower than the 1631.6s and 1870.2s of the SVM and FSVM models. Consistent with the findings of Leong et al. [26], the C-FSVM model outperforms the SVM model (80.33%), the FSVM model (87.57%), and the LSFSVM model (90.03%) in terms of overall sample classification accuracy. It is clear that the C-FSVM model is superior since it incorporates a variable penalty component on the basis of adding fuzzy membership degrees, which is a perfect fit for the real-world scenario of differential credit losses among institutions.

## Conclusion

Smart money management has entered the scene. Despite technological advancements, fundamental facets of finance remain unchanged; this includes the insidious nature of hidden, contagious, and unexpected financial dangers as well as the inability to control their spread. Financial risk assessments look at the supply chain as a whole and not just at any one link in it to determine risk. Financial institutions no longer solely base their risk assessment on the industry, firm size, and guarantee procedures associated with the applying SMEs. Supply chain finance, as a financial service for the entire supply chain, necessitates an evaluation of the overall industry risk faced by SMEs.

This study investigates the potential of technology to facilitate the creation of a model for assessing the financial risks inherent in the supply chain. A new, flexible penalty component is introduced to fine-tune the rating system based on the FSVM model. Finally, the empirical risk and confidence interval can be balanced with the help of the non-negative penalty factor C, which allows SVM model classification to incur the least amount of structural risk possible. The effectiveness of the C-FSVM risk assessment model is evaluated in this study by applying it to the Chinese automobile industrial sector. The total sample classification accuracy of the C-FSVM model is found to be 96.35%, while the identification accuracy rates for credible firms are 96.45% and for default enterprises they are 95.34%. It is evident that the C-FSVM risk assessment model developed here can be widely applied in commercial bank risk assessment practice. This study is not without its flaws. In this case, for instance, the research object is the automotive sector, which imposes constraints. There is a need for additional validation of the risk assessment model made possible by blockchain technology.

## Supporting information

S1 Data (ZIP)

## References

[1] Xu X, Chen X, Jia F, et al. Supply chain finance: A systematic literature review and bibliometric analysis. International Journal of Production Economics, 2018, 204: 160–173.

[2] Zhu Y, Zhou L, Xie C, et al. Forecasting SMEs’ credit risk in supply chain finance with an enhanced hybrid ensemble machine learning approach. International Journal of Production Economics, 2019, 211: 22–33.

[3] Chen J, Cai T, He W, et al. A blockchain-driven supply chain finance application for auto retail industry. Entropy, 2020, 22(1): 95. doi: 10.3390/e22010095 33285870 PMC7516533

[4] Baryannis G, Validi S, Dani S, et al. Supply chain risk management and artificial intelligence: state of the art and future research directions. International Journal of Production Research, 2019, 57(7): 2179–2202.

[5] Abdel-Basset M, Mohamed R. A novel plithogenic TOPSIS-CRITIC model for sustainable supply chain risk management. Journal of Cleaner Production, 2020, 247: 119586.

[6] Munir M, Jajja M S S, Chatha KA, et al. Supply chain risk management and operational performance: The enabling role of supply chain integration. International Journal of Production Economics, 2020, 227: 107667.

[7] DuHadway S, Carnovale S, Hazen B. Understanding risk management for intentional supply chain disruptions: Risk detection, risk mitigation, and risk recovery. Annals of Operations Research, 2019, 283: 179–198.

[8] Kouvelis P, Zhao W. Who should finance the supply chain? Impact of credit ratings on supply chain decisions. Manufacturing & Service Operations Management, 2018, 20(1): 19–35.

[9] Yazdani M, Gonzalez E D R S, Chatterjee P. A multi-criteria decision-making framework for agriculture supply chain risk management under a circular economy context. Management Decision, 2021, 59(8): 1801–1826.

[10] Zimon D, Madzík P. Standardized management systems and risk management in the supply chain. International Journal of Quality & Reliability Management, 2020, 37(2): 305–327.

[11] Ivanov D, Dolgui A. A digital supply chain twin for managing the disruption risks and resilience in the era of Industry 4.0. Production Planning & Control, 2021, 32(9): 775–788.

[12] Colicchia C, Creazza A, Menachof DA. Managing cyber and information risks in supply chains: insights from an exploratory analysis. Supply Chain Management: An International Journal, 2019, 24(2): 215–240.

[13] Xu S, Zhang X, Feng L, et al. Disruption risks in supply chain management: a literature review based on bibliometric analysis. International Journal of Production Research, 2020, 58(11): 3508–3526.

[14] Zekhnini K, Cherrafi A, Bouhaddou I, et al. Supply chain management 4.0: a literature review and research framework. Benchmarking: An International Journal, 2021, 28(2): 465–501.

[15] de Araújo Lima P F, Crema M, Verbano C. Risk management in SMEs: A systematic literature review and future directions. European Management Journal, 2020, 38(1): 78–94.

[16] Kusi-Sarpong S, Gupta H, Sarkis J. A supply chain sustainability innovation framework and evaluation methodology. International Journal of Production Research, 2019, 57(7): 1990–2008.

[17] Ghadge A, Weiß M, Caldwell ND, et al. Managing cyber risk in supply chains: A review and research agenda. Supply Chain Management: An International Journal, 2020, 25(2): 223–240.

[18] Ghadge A, Weiß M, Caldwell ND, et al. Managing cyber risk in supply chains: A review and research agenda. Supply Chain Management: An International Journal, 2020, 25(2): 223–240.

[19] Pettit TJ, Croxton KL, Fiksel J. The evolution of resilience in supply chain management: a retrospective on ensuring supply chain resilience. Journal of Business Logistics, 2019, 40(1): 56–65.

[20] Qian X, Papadonikolaki E. Shifting trust in construction supply chains through blockchain technology. Engineering, Construction and Architectural Management, 2021, 28(2): 584–602.

[21] Lezoche M, Hernandez JE, Díaz M M E A, et al. Agri-food 4.0: A survey of the supply chains and technologies for the future agriculture. Computers in industry, 2020, 117: 103187.

[22] Khan S, Khan MI, Haleem A, et al. Prioritising the risks in Halal food supply chain: an MCDM approach. Journal of Islamic Marketing, 2022, 13(1): 45–65.

[23] Adhi Santharm B, Ramanathan U. Supply chain transparency for sustainability–an intervention-based research approach. International Journal of Operations & Production Management, 2022, 42(7): 995–1021.

[24] Manavalan E, Jayakrishna K. A review of Internet of Things (IoT) embedded sustainable supply chain for industry 4.0 requirements. Computers & Industrial Engineering, 2019, 127: 925–953.

[25] Cole R, Stevenson M, Aitken J. Blockchain technology: implications for operations and supply chain management. Supply Chain Management: An International Journal, 2019, 24(4): 469–483.

[26] Leong WC, Bahadori A, Zhang J, et al. Prediction of water quality index (WQI) using support vector machine (SVM) and least square-support vector machine (LS-SVM. International Journal of River Basin Management, 2021, 19(2): 149–156.

